# Uncovering Forensic Evidence: A Path to Age Estimation through DNA Methylation

**DOI:** 10.3390/ijms25094917

**Published:** 2024-04-30

**Authors:** María Josefina Castagnola, Francisco Medina-Paz, Sara C. Zapico

**Affiliations:** 1 Department of Chemistry and Environmental Sciences, New Jersey Institute of Technology, Tiernan Hall 365, Newark, NJ 07102, USA; mc997@njit.edu (M.J.C.); fm368@njit.edu (F.M.-P.); 2 Department of Anthropology and Laboratories of Analytical Biology, National Museum of Natural History, MRC 112, Smithsonian Institution, Washington, DC 20560, USA

**Keywords:** age estimation, DNA methylation, biomarkers, forensic science, anthropology, crime scene

## Abstract

Age estimation is a critical aspect of reconstructing a biological profile in forensic sciences. Diverse biochemical processes have been studied in their correlation with age, and the results have driven DNA methylation to the forefront as a promising biomarker. DNA methylation, an epigenetic modification, has been extensively studied in recent years for developing age estimation models in criminalistics and forensic anthropology. Epigenetic clocks, which analyze DNA sites undergoing hypermethylation or hypomethylation as individuals age, have paved the way for improved prediction models. A wide range of biomarkers and methods for DNA methylation analysis have been proposed, achieving different accuracies across samples and cell types. This review extensively explores literature from the past 5 years, showing scientific efforts toward the ultimate goal: applying age prediction models to assist in human identification.

## 1. Introduction

### 1.1. Epigenetics

Epigenetics, derived from the Greek “epi” (ἐπί) meaning “upon” or “above,” refers to the chemical alterations of DNA that regulate gene expression without modifying the DNA sequence itself. Epigenetic modifications include DNA methylation, histone modification, chromatin remodeling, and non-coding RNAs. Particularly, DNA methylation has been related to different processes such as embryonic development, cellular reprogramming, transcriptional regulation, genomic imprinting, chromosomal stability, and X-chromosome inactivation (reviewed [1]). Nowadays, understanding this epigenetic modification has become crucial across fields such as forensic science and research related to aging and disease, prompting extensive investigation.

DNA methylation (DNAm) is a chemical modification where methyltransferases add a methyl group (-CH3) to the 5′ carbon of cytosines followed by guanines in a 5′-3′ direction in the DNA. In mammals, 60–90% of CpGs are methylated, while unmethylated regions are clustered into “CpG islands”. These islands are predominantly located at the promoters of housekeeping genes and consist of high-density CG content (>55%), typically ranging from 300 to 3000 base pairs in length [2]. DNA methylation is considered one of the most promising biomarkers for age estimation studies: as individuals age, specific CpGs become hypermethylated (gain of methylation) or hypomethylated (loss of methylation) [3]. These modifications can influence the activation or deactivation of the gene at certain sites and times, regulating protein production and affecting the individual’s observable features (phenotype).

Epigenetic alterations such as DNA methylation are part of the “hallmarks of aging”, representing shared processes among different organisms related to aging [4]. So far, twelve hallmarks of aging have been identified [5], which are closely interconnected and have been considered to enhance age estimation. Research on telomere length correlation [6], deletion in the mitochondrial genome [7], aspartic acid racemization [8], rearrangement in T-cells [9], accumulation of advanced glycation end products (AGEs) [10,11], and mRNA profile analysis [12] has been extensively conducted, showing different accuracies. However, each of these methods has shown specific limitations when used independently. In overcoming these challenges, DNA methylation has emerged as a promising hallmark for age estimation.

Specific CpGs can be chosen to gauge the rate at which an individual ages using what are known as epigenetic clocks [13,14,15]. Horvath defined an epigenetic clock as “the age estimate in years resulting from a mathematical algorithm based on the methylation state of specific CpGs in the genome” [16]. Thanks to epigenetic clocks, it is now known that DNA methylation patterns undergo significant changes during childhood, characterized by rapid accumulation followed by a stabilization phase in adulthood [14,17]. Moreover, the distinction between biological and chronological age was of special relevance to these studies. Chronological age refers to the calendar time that has passed since birth while biological age is a more ambiguous concept that depends on the aging process, mainly related to the relationship between the environment and the individual and how it impacts the phenotype. Hence, it is also referred to as physiological age, organismal age, or phenotypic age [16]. It is commonly observed that an individual’s biological age may not correspond to their chronological age [14,17,18] due to various factors such as environmental exposures, lifestyle habits, and diseases [19,20,21,22,23,24,25,26]. Moreover, factors like ancestry [27,28,29] and biological sex [13,24,30,31] could further contribute to this discrepancy.

### 1.2. DNA Methylation for Forensic Science

DNA methylation becomes of particular interest to the forensic sciences both in the context of criminalistics and forensic anthropology [32,33,34]. In the former case, estimating an individual’s characteristics from a biological sample found at a crime scene when there is no reference sample to compare with would help narrow down the pool of suspects. Furthermore, in forensic anthropology, age estimation is part of the biological profile for the identification of human remains. 

Age estimation is a component of Forensic DNA Phenotyping (FDP), which involves predicting a person’s externally visible characteristics (EVCs) alongside appearance and biogeographical ancestry. Examples include the extensively researched study of single nucleotide polymorphisms (SNPs) predictive for eye, hair, skin color, and biogeographical ancestry [35,36], which has become the most accurate and widely used in Forensic DNA Phenotyping. However, common SNPs proved inadequate for estimating certain individual characteristics that epigenetics could potentially address. Hence, in recent years, DNA methylation has been studied in forensic sciences for predicting chronological age [37,38,39], differentiating monozygotic twins [40,41], and identifying tissues and cell types [42,43].

In the realm of distinguishing monozygotic twins, who share identical genetic bases, conventional forensic DNA profiling falls short in providing differentiation. Nevertheless, they may display distinct DNA methylation patterns influenced by lifestyle factors, offering a new avenue for their individual identification. Although definitive markers for differentiation have still to be established, several studies employing various technologies present promising findings [40,41,44,45,46]. 

Another objective of DNA methylation in forensics is to identify the type of body fluid. In crime scenes, it is possible to find samples of unknown origin or complex mixtures, such as those in cases of sexual assault. In recent years, several research groups have explored both approaches as the differentiation between tissues could be immensely helpful in reconstructing a criminal case [47,48,49,50,51].

DNA methylation holds significant promise for the development of reliable, accurate, and practical methods for age estimation in the future. Identifying the most informative and sensitive markers, while considering the impact of various factors, is essential for integrating age prediction into routine forensic workflows. Over the years, the epigenetic age predictor most widely studied in relation to DNA methylation is the gene ELOVL2 (Elongation of Very Long Chain Fatty Acids Protein 2). Beginning with the studies of Garagnani et al. [52] in 2012, it remains a marker of great utility in forensic epigenetic age prediction. This is not only due to its high correlation with age across numerous tissues, but also, as emphasized by Aliferi et al. [53], because of its large methylation changes over the human lifespan. ELOVL2 has been extensively investigated in blood [54,55,56], teeth [57,58,59], bones [60,61,62], and buccal swabs [63,64,65], consistently demonstrating strong positive correlations with age. Age estimation models based solely on ELOVL2 CpGs have been developed [66,67]. Nevertheless, currently, the use of a single marker is not sufficient to achieve the accuracy required for age prediction models.

The challenges encountered in DNA methylation analysis are diverse and varied. As emphasized by Montesanto et al. [68], an ideal age prediction system within the forensic field should exhibit specific properties, including applicability to different tissues, replication across diverse populations, coverage of the entire age spectrum, and reproducibility across various technology platforms. The validation of these systems by multiple researchers will be critical for driving future advancements. This review delves into the recent progress in utilizing DNA methylation for age prediction within the field of forensic science.

## 2. Methods for Age Estimation by DNA Methylation Analysis

Over time, researchers have investigated various technologies to identify the most accurate DNA methylation markers for age prediction. These methods have targeted tissues commonly employed in forensic analyses, yielding heterogeneous outcomes. The choice of techniques hinges on specific objectives. When screening hundreds of loci, DNA microarray technology is required. However, for routine age prediction in forensic scenarios, the primary goal is to utilize a minimal number of DNA markers that enable analysis with low DNA input while maintaining sensitivity and accuracy.

**Figure 1:**
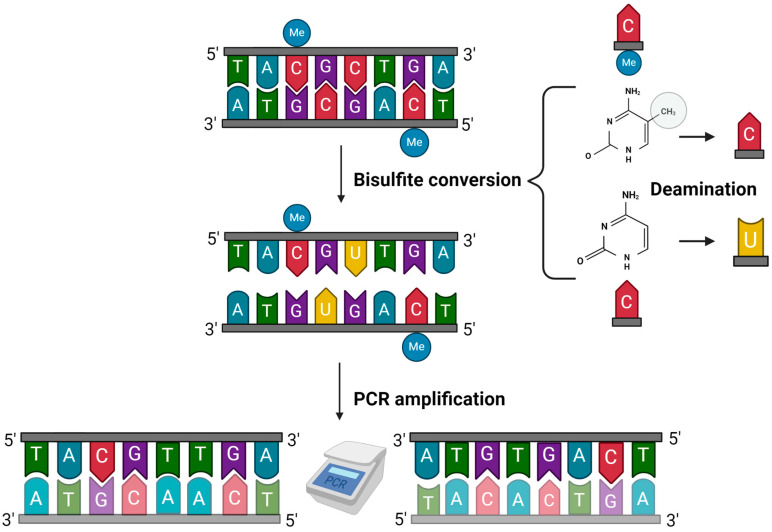
Bisulfite Conversion and Subsequent PCR Amplification. Sodium bisulfite deaminates unmethylated cytosines, converting them to uracil; methylated cytosines remain unchanged. During PCR amplification, uracils are replaced by thymines, allowing for comparison between the methylated and unmethylated strands. Created with BioRender.com.

Bisulfite conversion stands as the gold standard for DNA methylation analysis, with most of the current methods relying on it as the initial step for identifying CpG sites [69,70]. In this treatment, during the process of deamination, unmethylated cytosines are transformed into uracil, while methylated cytosines remain unaltered. As a result, the distinction between methylated and unmethylated cytosines can be observed as a change in the DNA sequence [70] (Figure 1). The limitations of bisulfite conversion are well-documented, primarily due to its tendency to cause significant DNA degradation, thereby reducing the amount of DNA available for subsequent analysis and lowering sequence information. Moreover, incomplete conversion of the sample and reannealing conversion are important considerations. Additionally, the quality of the DNA plays a crucial role in successful bisulfite conversion, which can be a limitation, particularly in forensic scenarios (reviewed [71]). Despite its challenges, bisulfite conversion has been fundamental in advancing the field of DNA methylation analysis. Multiple commercial kits are available for conducting DNA bisulfite conversion, and their performances have been assessed by different authors [72,73,74]. 

### 2.1. Bisulfite Sequencing by Sanger

Bisulfite sequencing is one of the oldest techniques for DNA methylation analysis. First, the sequence is treated by bisulfite conversion following PCR amplification. Then, the product is sequenced by the Sanger reaction. By comparing the treated DNA sample with the untreated one, it is possible to infer if a CpG site was methylated or not. One of the advantages is that this method is easy and simple to perform in any forensic laboratory. However, it can become challenging when studying multiple sites simultaneously within the sequence, particularly in noisy sequencing scenarios. Additionally, careful primer design is essential as they must effectively bind to both the methylated and unmethylated strands [71]. The method’s application can be observed in the work of Correia Dias et al., which encompasses analysis involving blood, bones, and teeth [60,75].

### 2.2. Methylation-Specific PCR (MSP)

MSP is a PCR-based method used to assess methylation status. Initially, it was based on two distinct PCR reactions (one for the methylated strand and one for the unmethylated strand). Subsequently, the PCR products, treated previously with bisulfite, were subjected to electrophoresis analysis [76]. Recently, the technique has been adapted for potential semi-quantification using real-time PCR. The percentage of methylated reference (PMR) is derived from the threshold values of each sample. By utilizing a single primer pair for the target sequence and constructing a standard curve with diluted samples of known methylation levels, it is possible to determine the proportion of methylation in the region of interest. The method’s advantages include its simplicity, minimal requirement of genomic DNA, and utilizing equipment commonly found in forensic laboratories [77,78]. Limitations are the inability to simultaneously analyze multiple CpG sites [71], non-quantitative nature, low specificity, and lack of methylation information at the CpG resolution level [79]. Current studies conducted using this method include the works of Kondo et al. and Ogata et al. [77,78] on teeth.

### 2.3. Methylation-Sensitive High-Resolution Melting (MS-HRM)

MS-HRM is based on the comparison between methylated and non-methylated sites generating differential melting profiles [80]. Following bisulfite conversion and PCR amplification, the product is subjected to increasing temperatures in the presence of an intercalating agent that emits fluorescence upon DNA binding. At a specific temperature, the double DNA strands dissociate, leading to the release of the dye from the DNA and a subsequent decrease in fluorescence. The dissociation time relies on the complementary bonds between both strands: CG pairs are distinguished by three hydrogen bonds, whereas AT pairs possess only two; consequently, the latter will dissociate faster than the former. After bisulfite treatment, methylated strains will exhibit a higher CG content, while non-methylated ones will show a higher AT content. Controls with known methylation levels allow comparison with the study sample in a graph of signal percentage over time. Finally, the derivative curve will reveal melting peaks, with the peak with non-methylated sites appearing to the left (at lower melting temperatures) compared to methylated sites (at higher melting temperatures). Primers must be designed to amplify both types of strands; however, often there is a differential amplification towards the non-methylated strand, a phenomenon known as PCR bias. Several strategies to mitigate this issue, such as optimizing primer design and PCR conditions, have been described in the literature [80,81,82].

MS-HRM is considered both cost-effective and time-efficient for assessing methylation at a single locus [71,80,83,84]. For this method, the obtention of a pure PCR product is crucial. However, one drawback is that although it is possible to determine the general methylation status of the region of interest, providing the status of a specific CpG is not feasible. Furthermore, conducting a multiplex with several primers targeting different regions would hinder the clear visualization of distinct peaks and increase the complexity of results interpretation. This presents another disadvantage, particularly considering that age estimation currently achieves greater accuracy with the use of multiple markers. This review includes several examples of this method in blood samples [83,85], saliva [84,85,86], and semen [85].

### 2.4. MassArray (EpiTYPER)

EpiTYPER^®^ utilizes mass spectrometry for quantitative analysis of DNA methylation on a large scale. Following PCR amplification of bisulfite-converted DNA, reverse strand transcription is performed, and the resulting RNA product is subjected to cleavage after each U base. The resulting fragments are then analyzed using a mass spectrometer (MALDI-TOF), yielding different masses according to the composition of the DNA strand. Analysis of the peaks in the spectrum allows differentiation by weight of methylated versus unmethylated cytosines, generating a methylation ratio at each location in the sequence [87].

The method offers advantages in terms of speed, accuracy, and ability to analyze a large number of samples. However, it faces limitations related to cost-effectiveness in high-throughput analysis, potential misinterpretations of methylation levels due to polymorphisms, and the presence of contaminant peaks in the analysis [71]. Several studies using EpiTYPER are mentioned in the following sections [58,88,89,90].

### 2.5. Multiplex Minisequencing Reaction (SNaPshot) 

The SNaPshot assays present a semi-quantitative technique for evaluating DNA methylation, employing dideoxy single-base extension (SBE) with capillary electrophoresis. After the PCR amplification of bisulfite-converted DNA, a SBE reaction is performed to analyze a nucleotide change occurring at a specific CpG within the region of interest. The nucleotide to be extended following the SBE primer is fluorescently labeled, and depending on which nucleotide binds to the complementary strand, the color of the peak detected by the capillary electrophoresis instrument will differ. The peak’s intensity is correlated with the level of methylation. For each SBE primer, only one CpG can be analyzed. A major advantage is its ability to conduct a multiplex reaction targeting various regions, thereby expanding the analysis scope. Additionally, the instrumentation employed is capillary electrophoresis, commonly utilized for STR analysis in forensic laboratories [91,92]. A potential disadvantage of this method is that the development of a multiplex system might be time-consuming and less straightforward. Furthermore, due to its semi-quantitative nature, this method might not offer the level of precision necessary for in-depth DNA methylation analysis.

Numerous SNaPshot multiplex assays have been designed for age prediction using forensically significant tissues such as blood [60,64,93,94,95], saliva [37,96], semen [97], and bones and teeth [60]. 

### 2.6. Pyrosequencing

Pyrosequencing is considered the gold standard technique for the identification of allele-specific methylation patterns [98]. This method relies on chemiluminescence detection to determine the sequence of interest, previous bisulfite conversion and PCR amplification. It operates as a sequencing-by-synthesis technique, where deoxynucleotide triphosphates (dNTPs) are each dispensed into a chamber containing the DNA template. When the correct complementary dNTP is added by the polymerase, inorganic pyrophosphate (PPi) is released. Through enzymatic reactions, the PPi generates light, observed as sequential peaks in a pyrogram. The height of these peaks correlates with the proportion of pyrophosphate released, indicating the number of nucleotides added. Thus, the quantity of cytosines and thymines at a particular position can be determined by comparing the peaks, thereby revealing the level of methylation [71,99,100]. It is important to highlight that fluorescence in pyrosequencing is produced by nucleotide incorporation during PCR, whereas in Sanger sequencing, it is determined by nucleotide chain termination [100,101].

Pyrosequencing is a quantitative technique, which makes it very useful in forensics. Its primary advantages include its relative simplicity, high reproducibility, capability to discern differences of less than 5% in methylation levels, and its applicability for heterogeneous samples [71,102]. Compared to massively parallel sequencing (discussed in the following section), this method has restricted multiplexing capability [103]. Furthermore, even with its excellent quality-to-price ratio [71], and its practical use in routine forensic cases remains limited. However, for research purposes, pyrosequencing is widely used, and examples can be observed in multiple sources: blood and bloodstains [56,57,67,104], saliva and buccal swabs [65,85,105], semen [85,106], and teeth [59,107,108].

### 2.7. Next Generation Sequencing (NGS)

NGS, also known as massively parallel sequencing (MPS), is a high-throughput DNA sequencing method where billions of short reads are sequenced per instrument run. NGS has significant advantages for analyzing a wide range of specific methylation sites within a single reaction, enabling extensive exploration of genetic information. There are different NGS platforms, each with their own technology and distinctive characteristics. In epigenetics, for example, it is common to find platforms based on sequencing by synthesis like Illumina^®^ BeadChip arrays (San Diego, CA, USA). Different approaches can be identified, either using a large number of markers across the entire genome (whole-genome sequencing) or focusing on exons (whole-exome sequencing), as well as the analysis of a small number of CpG markers (targeted sequencing) [109]. In the literature, there are both examples of whole-genome sequencing (WGS) [37,52,110,111] studies and targeted NGS approaches [38,112,113,114]. Many of these studies belong to the VISAGE Consortium (VISible Attributes through GEnomics), which has emerged in recent years as a tool that employs NGS to create and validate models for predicting appearance, ancestry, and age. NGS enables thorough screening to identify potential new DNA methylation markers, which can then be used by the same method to develop prediction models with a smaller subset of candidates. 

The advantages of this method include the simultaneous analysis of a large number of DNA markers in a very short period of time and the obtention of high-resolution data. Moreover, it possesses the capability to process low quality/quantity DNA, a crucial advantage given the prior bisulfite conversion step and forensic contexts [53]. The disadvantages include the elevated equipment/infrastructure costs and the complexity of the data analysis, necessitating thorough training for laboratory personnel in NGS data processing [115].

### 2.8. Exploring New Approaches in DNA Methylation Analysis

Droplet Digital PCR (ddPCR) is an innovative quantitative method. In the first steps, the sample is fractionated into thousands of microdroplets of bisulfite-converted DNA, followed by PCR amplification and analysis of each droplet. This enables parallel digital sequencing of single molecules. The method is highly sensitive and rapid [71,116]. Furthermore, in comparison to traditional qPCR, ddPCR is less dependent on PCR inhibition or high PCR efficiency [117] and could be a more efficient procedure due to its simplicity in a single PCR amplification [118]. However, ddPCR requires specialized instrumentation and primer design can be labor-intensive [71]. In forensics, there are examples of this method in blood samples [116,119,120] and saliva samples [121]. 

Enzymatic-based non-chemical conversion techniques are being investigated as alternatives to bisulfite conversion. Vaisvila et al. introduced an enzymatic methylation sequencing (EM-seq) method capable of detecting methylated and non-methylated cytosines using sets of enzymatic reactions [122]. However, further studies are needed for its extensive use, and bisulfite conversion remains the method of choice for DNA methylation analysis in forensic research.

**Figure 2:**
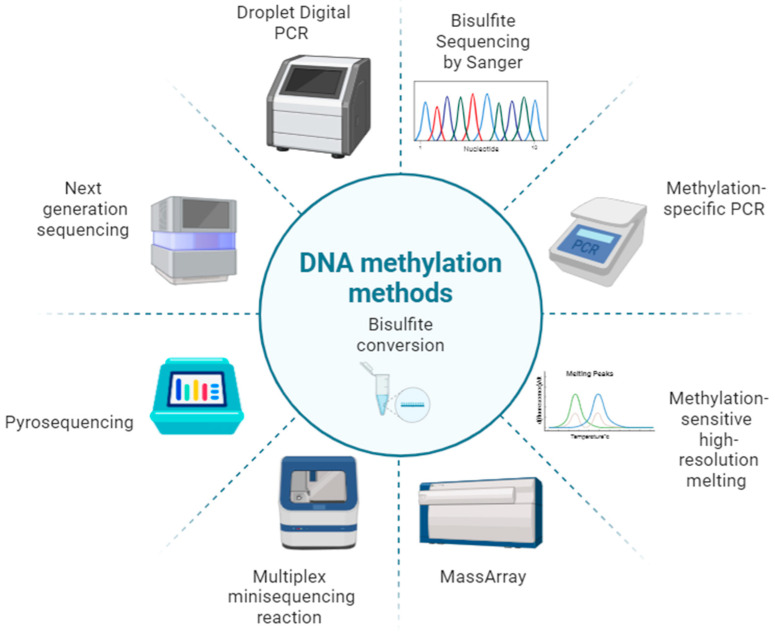
Methods for estimating age by DNA methylation analysis. Each workflow begins with bisulfite conversion as the initial step. Created with BioRender.com.

In summary, a range of methods have been explored for age prediction based on DNA methylation (Figure 2). The outcomes vary depending on their specific strengths and limitations. Bisulfite conversion is commonly employed as the initial step in the analysis and so, it is critically important to monitor conversion efficiency and variations in performance across different kits [123]. Technical errors in DNAm analysis can vary across analysis technologies, emphasizing the importance of conducting training, testing, and validation of models using the same technology to integrate them into routine forensic workflows [124]. Furthermore, there are instances where reference data produced from a particular DNAm microarray technology is subsequently utilized in forensic analyses employing a different technology, leading to variations in the results [103]. Correction models, like the one introduced by Feng et al. [88], which utilizes Z-score transformation to address differences between reference model data generated from EpiTYPER microarrays and actual casework data produced with pyrosequencing, are pivotal in managing these variations.

## 3. Epigenetic Clocks

Epigenetic clocks originated from genome-wide studies that analyzed DNA methylation patterns at specific genomic positions [13,14,125]. These investigations explored how methylation changes with age and its association with factors such as biological sex, disease status, and lifestyle choices (including smoking, diet, exercise, and alcohol consumption). As previously mentioned, epigenetic clocks introduced the concepts of chronological age (measured in calendar time since birth) and biological age (dependent on an individual’s biological state) [16]. In forensic science, the primary objective is to estimate an individual’s chronological age, especially in criminal investigations or for anthropological purposes. However, when the focus shifts to understanding how environmental factors influence phenotypes and contribute to human aging, researchers delve into biological age (reviewed [126]).

Chronological clocks focus exclusively on CpG sites that correlate with chronological age, whereas biological clocks encompass a broader range of CpG sites associated with factors such as lifestyle and lifelong environmental influences. The term age acceleration residual (AAR) refers to the discrepancy between predicted age and actual chronological age. Elevated AAR has been associated with a higher risk of mortality [21,127,128]. Ideally, a perfect chronological clock would yield zero AAR. However, distinguishing between chronological and biological components of aging remains a significant challenge (reviewed [126]).

Chronological clocks are considered first-generation, exemplified by Hannum et al. and Horvath clocks, which have achieved remarkable accuracy with correlation coefficients exceeding 0.9. Hannum et al. examined over 450,000 CpG markers in whole-blood human samples (ranging from 19 to 101 years old) and identified 71 methylation markers strongly associated with age. However, Hannum’s clock loses accuracy when applied to non-blood tissues and samples from children [13]. 

Horvath developed a multi-tissue clock composed of 353 CpGs, using Illumina DNA methylation array datasets and samples from 51 healthy tissues and cells of young children and adults [14]. A total of 193 CpGs were identified as positively correlated with age, while the remaining 160 CpGs showed a negative correlation. The pace of these DNA methylation changes accelerated during growth and development. While the methylation status of individual CpGs showed weak associations with age, combining the 353 CpGs yielded a robust biomarker of biological aging. Thus, a higher number of CpGs enhanced accuracy and robustness. Moreover, within the same study, the analysis of cancer samples showed significant age acceleration. 

The second generation of epigenetic clocks consists of biological clocks, designed to improve assessments related to factors like time to death and healthspan [129]. Zhang et al. were the first to integrate mortality-associated CpGs to create an overall mortality risk score [130]. However, a more robust predictor emerged later: PhenoAge [131]. A total of 42 clinical biomarkers were assessed using blood samples in a cohort of 9926 individuals, considering factors such as creatine, C-reactive protein, white blood cell count (WBC), and other indicators to develop an age prediction model composed of 513 CpGs. Out of these, only 41 markers overlapped with Horvath’s clock, with five being shared among Hannum et al., Horvath, and Levine et al.’s epigenetic clocks [13,14,131]. The CpGs shared among these clocks demonstrated a stronger correlation with chronological age, whereas the non-shared CpGs were more indicative of biological age. This observation supports the notion that the initial generation of DNAm age estimators was primarily linked to chronological age and exhibited fewer associations with clinical measures of biological age, as seen in PhenoAge [132,133]. This biological clock surpassed the initial generation of DNAm clocks in predicting various health outcomes, including all-cause mortality, cancers, healthspan, cardiovascular disease, and Alzheimer’s disease [131]. 

In addition to PhenoAge, scientists have subsequently introduced another biological clock known as GrimAge [129]. This epigenetic clock was developed in correlation with biomarkers for 12 of the plasma proteins, chronological age, biological sex, and smoking (measured in smoking pack-years), to predict the time to death [126]. Using large-scale validation data from three ancestry groups, the age acceleration measure (AgeAccelGrim) surpassed former epigenetic clocks in predicting time-to-death, time-to-coronary heart disease, and time-to-cancer, and was linked to computed tomography data for fatty liver/excess fat and early age at menopause. It also strongly correlated with comorbidities, exhibiting associations with lifestyle factors like a healthy diet and educational attainment. Notably, GrimAge has been used to study many conditions including COVID [134], autism [135], major depression disorder [136], and post-traumatic stress disorder (PTSD) [137].

The second version of GrimAge (GrimAge 2) used two additional DNAm-based estimators of plasma proteins: high-sensitivity C-reactive protein (logCRP) and hemoglobin A1C (logA1C) [138]. GrimAge2 was assessed in 13,399 blood samples from nine study cohorts, which included individuals of Hispanic, European, and African populations (aged 40 to 92 years). This second version outperformed GrimAge in predicting mortality and exhibited stronger associations with age-related conditions, including kidney and lung dysfunction, metabolism, cognitive behavior, lipid profiles, vital signs, and CT-derived measures of adiposity across multiple racial and ethnic groups. Regarding DNAm markers of metabolic syndrome, DNAm logCRP was positively correlated with morbidity count, and DNAm logA1C was highly associated with type 2 diabetes. GrimAge version 2 was also studied in younger individuals and saliva samples, extending the analysis beyond the initial version.

While Hannum’s and Horvath’s epigenetic clocks used different CpGs to predict actual age-related to all-cause mortality, PhenoAge and GrimAge used CpG methylation to improve the previously proposed age-related mortality and phenotypic indicators, adjusted for chronological age [139]. Both first-generation and second-generation epigenetic clocks mentioned earlier offered a cross-sectional view, capturing an individual’s methylome at a specific point in time [126]. Emerging longitudinal methylation studies, such as DunedinPoAm and DunedinPACE, enabled exploration of methylation changes over an extended period, showing how epigenetic modifications evolve in individuals over time. 

DunedinPoAm (Dunedin Pace of Aging methylation) evaluated the rate of biological aging using whole-genome blood DNA methylation data and elastic-net regression [140]. This epigenetic clock analyzed differences in biological aging rates among 954 individuals who shared the same birth year and followed changes in 18 biomarkers indicating organ-system integrity during 12 years. Higher DunedinPoAm scores correlated with midlife cognitive and physical decline, accelerated facial aging, and increased risk of disease and mortality in older adults. Among young individuals, experiences of early-life adversity were also associated with a faster DunedinPoAm. Furthermore, the study included validation analysis conducted within cohort studies and the CALERIE trial.

The same research team subsequently developed DunedinPACE (Dunedin for Pace of Aging Calculated from the Epigenome) as a DNA-methylation biomarker to quantify the pace of aging through a blood test [141]. Using data from a cohort of people of the same chronological age, it tracked the within-individual decline in 19 indicators of organ system integrity over a two-decade period. It had three distinguishing features: it analyzed a single-year birth cohort, conducted follow-ups in young adults to separate effects from disease effects and avoid survival bias, and focused on changes in multi-organ system integrity during adulthood to distinguish ongoing aging processes from early developmental deficits. DunedinPACE demonstrated correlations with morbidity, disability, and mortality, and identified accelerated aging among young adults with a history of childhood adversity, providing information on how behavioral and environmental modifications may influence the rate of aging. One limitation of DunedinPACE is that it was established in a small cohort from a single country and did not consider individual diseases or causes of death. Assessing it in larger datasets would enable researchers to explore further the impacts of specific diseases or causes of death. 

The continuous evolution and improvement of epigenetic clocks can be beneficial both in forensic contexts and for enhancing human health. Considering the features observed thus far, selecting the appropriate epigenetic clock based on the need to estimate chronological or biological age is essential.

## 4. DNA Methylation Analysis for Anthropology

Precise age estimation plays a critical role in anthropological investigations, especially when reconstructing the biological profiles of skeletal remains in scenarios like mass disasters, genocides, and bioarchaeology/paleodemography. 

The hardest structures in the human body, resistant to decomposition, include bones and teeth and are crucial for postmortem examinations, playing a significant role in forensic anthropology [142]. While accurately estimating age in children is feasible through anthropological markers of growth and development, the process becomes significantly more challenging in adults. Estimating age-at-death in adult individuals relies on assessing the degeneration of skeletal and dental structures, which involves examining macroscopic characteristics and evaluating anatomical features (reviewed [143]). Still, the difference between chronological and estimated age can be ±10 years, which could hamper the proper identification of the victim [59]. As a result, researchers have investigated a range of techniques based on the biochemical mechanisms of aging to improve the age-at-death estimation in adult individuals.

Among these techniques, aspartic acid racemization stands out as the oldest one [8,144,145], followed by protein glycosylation [146], telomere length measurement [108,147], mitochondrial mutations [148,149], DNA damage response [150], and T-cell DNA rearrangement [147], among others. Nonetheless, their widespread use in forensic sciences is hindered by limitations such as accuracy, applicability, or technical complexity. External factors like pathological conditions and mass disasters [151] significantly influence the subject of study. For instance, although aspartic acid racemization shows good accuracy with a mean absolute error (MAE) of 5 years [8], it is not suitable for analyzing burnt human remains due to temperature constraints [144]. Consequently, with the growing understanding of DNA methylation, it emerges as a promising biochemical biomarker for age prediction in anthropology. However, research regarding DNA methylation analyses in bone and tooth tissues has been relatively limited.

Bekaert et al. [57] pioneered the initial analysis of DNA methylation in teeth for age estimation. Their research focused on analyzing four genes (ASPA, PDE4C, ELOVL2, and EDARADD) using DNA extracted from 29 dentin samples (third molars) obtained from individuals aged between 19 and 70 years old. Through pyrosequencing, they established an age estimation model that yielded a mean absolute deviation (MAD) of 4.86 years.

Giuliani et al. [58] were the first to introduce age estimation techniques using a combination of different layers of tooth samples employing EpiTYPER. They presented unique age prediction models for each tooth tissue (dental pulp, cementum, and dentin) individually, as well as combined models. Each model incorporated 5–13 CpGs from the ELOVL2, FHL2, and PENK genes, showing MADs ranging from 1.2 to 7.1 years, depending on the tissue analyzed. Notably, the most accurate correlations were found when combining pulp and cementum (MAD = 1.2), and dental pulp alone (MAD = 2.25).

Márquez-Ruiz et al. [108] employed pyrosequencing to evaluate methylation levels at specific CpG sites in the ELOVL2, ASPA, and PDE4C genes for age prediction. Using 65 whole teeth samples from individuals aged 15 to 85 years, they achieved MAEs ranging from 4.8 to 6.9 years using the three genes. The study further explored the correlation between methylation data and relative telomere length measurements to develop age prediction quantile regression models for both biomarkers together and separately. Results indicated that DNA methylation was more informative than telomere length when evaluated independently, and the combined study suggested limited utility for telomere length as a supplementary marker with DNA methylation markers for age estimation. They found no significant impact on the age prediction based on the type of tooth or biological sex. The final estimation model was based on nine CpGs in two genes (ELOVL2 and PDE4C), resulting in a MAE of 5.04 years.

Zapico et al. [59] were the first to use DNA methylation to estimate age in pulp tissues by applying pyrosequencing. Employing a set of 20 healthy erupted third molars (age 22–70), researchers integrated established DNA markers from the ELOVL2 and FHL2 genes, alongside three newly identified ones (NPTX2, KLF14, and SCGN), subjecting them to analysis within four distinct multivariate regression models. The outcome demonstrated great accuracy, with MAEs ranging from 1.5 to 2.13 years when comparing predicted age to chronological age in adult individuals. Two important factors to consider are: firstly, that this study exclusively utilized third molars, which are protected within the jaw, potentially influencing the study’s outcomes; secondly, the use of pulp may also significantly impact the accuracy of age estimation, as its location and properties render it more resistant to environmental stresses.

Correia Dias et al. [75] developed two multi-tissue models for age estimation, employing 31 bones and 31 whole tooth samples and using both Sanger sequencing and SNaPshot techniques to analyze DNA methylation levels. For Sanger sequencing, the optimal model for bones included six CpGs located in the genes ELOVL2, EDARADD, and MIR29B2C, obtaining a MAD of 2.56. In the case of teeth, the marker FHL2 CpG 4 had the best performance, with a MAD of 11.35. When genes were evaluated with SNaPshot, the best model for bones included genes FHL2 and KLF14, producing a MAD of 7.2. For teeth, the optimal model included CpGs at ELOVL2 and KLF14, yielding a MAE of 7.1. In a follow-up study, Correia et al. [60] developed the first multi-age prediction models for bones and teeth. They built two multi-tissue models for age estimation utilizing Sanger sequencing and SNaPshot, incorporating blood, bones, and tooth samples from both living and deceased individuals.

Kondo et al. [77] developed the first age estimation method for teeth using real-time methylation-specific PCR (RT-MSP), focusing on the validated biomarker ELOVL2. They built a single-gene age prediction model using 29 whole teeth samples spanning ages from 20 to 79 years, achieving a MAD of 8.94. In this case, it is important to consider that only one marker was utilized, which may have impacted the accuracy. Moreover, they observed that methylation levels were not influenced by biological sex. Following this, Ogata et al. [78] extended the application of RT-MSP by incorporating a CpG site in the EDARADD gene alongside the previous marker in the ELOVL2 gene. In this instance, the samples were also whole teeth (n = 59), and they developed a multiple regression prediction model, achieving a MAE of 6.69 years. Validation of the age estimation model using an additional 40 teeth resulted in a MAE value of 8.28 years. Similar to the previous study, they highlighted the importance of further exploration of this method, especially given its affordability and accessibility in forensic laboratories.

In recent years, genome-wide DNA methylation data from bone samples has been generated in various publications [61,152]. The various CpG sites identified to predict age from these different datasets further contribute to the knowledge for the future development of a more accurate bone clock. In the realm of new technologies such as NGS, the VISAGE consortium developed age prediction models for various tissues, including bones [62]. The bone-specific model (n = 161) utilized six CpGs from four genes (ELOVL2, KLF14, PDE4C, and ASPA), achieving a MAE of 3.4.

The development of prediction models in bones and teeth is not as extensive as for blood or saliva samples, which is why more studies and additional validations are needed. In anthropology, samples are often in highly diverse conditions, so the impact of taphonomy and environment, as well as the selection of tissue and markers, are crucial. Furthermore, the development of new epigenetic clocks specific to teeth and bone would also be important for future advancements in the field.

### Age Estimation in Children

In childhood, age estimation through forensic anthropological assessment of growth and development can be achieved with high accuracy (reviewed [144]); however, there are situations where these techniques are limited. In cases of child abduction, missing children, and unaccompanied minors in migration, estimating age could be critical [143,153,154], and DNA methylation could become highly useful.

Among recent discoveries, Freire-Aradas et al. [90] conducted a search aimed at identifying potential DNA methylation markers correlated with age among blood donors aged 2 to 18 years (n = 209). First, public datasets from Illumina BeadChip arrays were analyzed, followed by the utilization of EpiTYPER DNA methylation analysis system to identify six loci (KCNAB3, EDARADD, ELOVL2, CCDC102B, MIR29B2CHG, CR_23_CpG_3) associated with age. Their novel finding was the strong correlation between age and the KCNAB3 gene (potassium voltage-gated channel subfamily A regulatory beta subunit 3, chromosome 17), showing rapid changes between the ages of 2 and 18 years. This underscores the potential of this gene as a biomarker for childhood and adolescence. 

McEwen et al. [155] then created a specialized epigenetic clock for children, using noninvasive buccal epithelial swab samples from people aged 0 to 19. The data, generated and evaluated from 1721 genome-wide DNA methylation profiles, was then employed to create the Pediatric Buccal Epigenetic (PedBE) clock, which estimates the age based on methylation patterns across 94 CpGs (MAE = 0.35). The PedBE clock has since been utilized in later publications for age estimation in children [156,157]. As buccal swabs are deemed noninvasive for collection, they could offer advantages, particularly in children.

Freire-Aradas et al. [89] studied the development of a new epigenetic clock that included children and adolescents. The prediction model used 895 DNA blood samples from people aged 2 to 104, employing the EpiTYPER technique. Through a comparison of various statistical methods, they identified the optimal prediction model as a quantile regression neural network applying markers from ELOVL2, ASPA, PDE4C, FHL2, CCDC102B, MIR29B2CHG, and chr16:85395429 (GRCh38). The validation model based on a quantile regression neural network exhibited the highest accuracy, with a MAE of 3.32 across 152 samples. One important aspect of this study was the comparison of multiple statistical methods, which demonstrated the advantage of the quantile regression tool in generating age-dependent prediction intervals, thereby enabling the adjustment of errors to match the estimated age.

Based on the gathered information, there is a need to delve deeper into the recently discovered markers for children. Additionally, novel prediction models should be generated using different techniques and tissues based on these recent epigenetic clocks. This would improve the identification of children and adolescents when the most recognized anthropological methods are not feasible to use.

## 5. DNA Methylation Analysis for Criminalistics

### 5.1. Blood

The initial epigenetic age prediction methods introduced in forensic science were mainly tailored for estimating age using blood samples, as it would not be uncommon, for instance, to encounter bloodstains at a crime scene. The most renowned DNA marker ELOVL2 was initially examined in blood samples by Garagnani et al. [52] using the Illumina HumanMethylation450 BeadChip array (San Diego, CA, USA), and subsequently validated in follow-up studies [13,67]. One of the early works on age prediction models using pyrosequencing was conducted by Weidner et al. [15]. This study analyzed 151 blood samples and focused on three CpG sites within the genes ITGA2B, ASPA, and PDE4C, obtaining a MAD of less than 5 years.

Zbieć-Piekarska et al. [67] evaluated the methylation status of seven CpGs within the ELOVL2 gene in 427 blood samples from Polish individuals using bisulfite pyrosequencing. Their final model included two CpG sites in ELOVL2 and enabled age prediction with a MAD of 5.75 in the test set (n = 124). Additionally, they analyzed the methylation levels in bloodstains to evaluate the stability of prediction accuracy over time, finding no changes after 4 weeks of room-temperature storage. The subsequent study conducted by Zbieć-Piekarska et al. involved the analysis of 41 CpGs in 420 blood samples (age 2–75 years) using pyrosequencing. The age prediction model, which utilized five markers from the genes ELOVL2, TRIM59, C1orf132/MIR29B2CHG, KLF14, and FHL2, yielded a standard error (SE) of the estimate of 3.9 in the test set (n = 120) [18]. Later, Cho [54] used this same development to construct multiple age prediction models in 100 blood samples from the Korean population. In this case, they employed the same genes from Zbieć-Piekarska et al.’s model, where the most accurate age prediction was achieved using six CpG sites across the genes ELOVL2, TRIM59, C1orf132/MIR29B2CHG, FHL2, excluding KLF14 (MAD = 3.29).

Several studies employed SNaPshot assays in different populations and markers. Jung et al. [64] used 448 samples from various tissues, including blood, to develop independent age prediction models and a combined one based on five CpG sites (genes ELOVL2, FHL2, KLF14, C1orf132/MIR29B2C, and TRIM59), achieving a MAD of 3.48 years for blood. Pan et al. [93] analyzed 310 blood samples from the Chinese Han population using a multiplex methylation SNaPshot assay. They incorporated seven CpG markers from genes ASPA, EDARADD, KLF14, CCDC102B, ZNF423, ITGA2B, KLF14, and FHL2 to construct two distinct age prediction models (stepwise regression and support vector regression). Notably, the support vector regression model was the most accurate, achieving a MAD of 5.56 in the test set (n = 80). Onofri et al. [94] aimed to study previous models [54,64] and validate them in an Italian population. They employed 84 blood samples in a SNaPshot assay targeting five CpG sites in the genes ELOVL2, FHL2, KLF14, MIR29B2C, and TRIM59, achieving a MAD of 3.01 years in the test set. 

Feng et al. [88] investigated 153 age-associated CpG sites within 21 genomic regions in 390 Chinese blood samples (aged 15–75 years) using the EpiTYPER system. Their primary objective was to determine the optimal feature selection method. In two independent validation sets, they identified nine CpG sites located in genes ELOVL2, TRIM59, MIR29B2CHG, PDE4C, CCDC102B, RASSF5, and a region on chr10:22334463/65 as the optimal subset for age estimation (MAD = 2.49). Notably, the linear model performed better than machine learning models like support vector machine (SVM) and artificial neural network (ANN). Additionally, they showed that a z-score transformation could partially remove the batch effect between data generated from EpiTYPER and pyrosequencing techniques.

In their study, Lau and Fung [110] analyzed DNA methylation from 991 blood samples (aged 19–101 years) using Infinium^®^ HumanMethylation450 BeadChip (San Diego, CA, USA). They explored various variable selection methods including forward selection (FS), least absolute shrinkage and selection operator (LASSO), elastic net (EN), and smoothly clipped selection deviation (SCAD), to predict human age. With this information, they compared the performance of classical statistical models (multiple linear regression) with sophisticated machine learning algorithms (random forest regression, one hidden layer, two hidden layers, and SVM). Their analysis revealed that the optimal model was achieved from the forward selection method of 16 CpG markers alongside the multiple linear regression statistical model, resulting in a MAD of 3.76. Notably, they found that increasing the number of markers beyond this threshold did not improve the model’s accuracy.

The VISAGE Consortium [158] developed a prototype tool for age estimation employing a multiplex PCR/MPS assay. They analyzed 32 CpGs from five genes (ELOVL2, MIR29B2C, FHL2, TRIM59, and KLF14), previously identified by Zbieć-Piekarska et al. [18], including reproducibility and sensitivity analysis, achieving robust quantification of methylation levels (mean standard deviation of 1.4% across ratios). Moreover, The VISAGE enhanced tool for age prediction in somatic tissues, incorporating six CpG sites from genes previously studied by Heidegger et al. [158] alongside PDE4C, yielded a MAE of 3.2 in the test set of 48 samples. In a subsequent study, the VISAGE consortium [62] developed a prediction model from blood samples (n = 160) using six CpGs from six genes (ELOVL2, MIR29B2CHG, KLF14, FHL2, TRIM59, and PDE4C), achieving a MAE of 3.2 years.

Multiple studies have explored age prediction using bloodstains, especially important in crime scene investigations [56,67,159,160]. The most recent study, Yang et al. [56] utilized pyrosequencing and random forest regression (RFR) to develop an age prediction model. Initially, they evaluated 46 CpG sites from six genes (ELOVL2, C1orf132, TRIM59, KLF14, FHL2, and NPTX2) using bloodstain samples from 128 males and 113 females (aged 10 to 79 years). Subsequently, they employed RFR to build two models, one for males (MAD = 2.8) and another for females (MAD = 2.93). A notable distinction is that they obtained reproducible results using only 0.1 ng of genomic DNA. 

Age estimation models in blood samples have been extensively studied in forensic research. Multiple studies have explored various techniques, markers, sample types, and statistical models, leading to varying levels of accuracy.

#### 5.1.1. Postmortem Blood Samples

In addition to age estimation from blood samples left at the crime scene, potentially belonging to the perpetrator, it is important to assess whether blood from a deceased individual could differ in methylation patterns affecting age estimates. The following provides a brief description of the work published to date on this topic.

The first study related to age prediction using DNA methylation in deceased samples was by Bekaert et al. [57]. They investigated DNA methylation patterns employing pyrosequencing technology targeting four CpG markers (ASPA, PDE4C, ELOVL2, and EDARADD). The model was built from blood samples of both living (n = 37) and deceased (n = 37) patients aged 0 to 91 years old, achieving a MAD of 3.75 years. Two important discoveries emerged from the study: prediction accuracy remained consistent across samples from both living and deceased individuals, and there were no discernible differences based on biological sex. These results are consistent with the findings of Hamano et al. [83], who analyzed blood samples from 22 living and 52 deceased individuals aged 0 to 95 years and developed a combined age prediction model. Using markers in the genes ELOVL2 and FHL2 through MS-HRM, they yielded a MAD of 7.71 years for the test set. They also included the information that the samples were analyzed within 10 days after death, suggesting that these data could be important for future comparisons when establishing a prediction model.

Correia Dias et al. [161] performed bisulfite Sanger sequencing on blood samples obtained from 51 deceased individuals (24 to 86 years), processed within 5 days postmortem. They evaluated the methylation levels of ELOVL2, FHL2, EDARADD, PDE4C, and C1orf132, reporting a MAD of 6.08 years for the training set and 8.84 years for the test sets. The same group [95] developed prediction models using blood samples from 59 living and 62 deceased individuals (28 to 86 years) utilizing SNaPshot assays and building upon CpG sites analyzed in a previous study [64]. For the final model applied to living individuals, they employed three CpG sites located at the ELOVL2, FHL2, and C1orf132 genes, resulting in a MAD of 4.25 years. In contrast, for the final model in deceased individuals, they integrated four CpG sites found in the ELOVL2, FHL2, C1orf132, and TRIM59 genes, yielding a MAD of 5.36 years. Similar to previous studies, they found no differences in prediction accuracy based on biological sex.

Anaya et al. [104] employed bisulfite pyrosequencing, similar to Bekaert’s previous work [57], to assess individual CpG sites on five genes (KLF14, ELOVL2, C1orf132, TRIM59, and FHL2) in 264 postmortem blood samples ranging from 3 months to 93 years of age, achieving a MAD of 7.42 for the testing data (n = 72). Furthermore, the researchers explored potential factors that could influence accuracy. These factors included sample storage time before analysis, which in this case ranged from 2.5 to 4 days. Additionally, a lower prediction potential of age estimation as an individual’s age increases, consistent with prior research [57].

Naue et al. [38] employed MPS to investigate 13 previously selected CpGs (DDO, ELOVL2, F5, GRM2, HOXC4, KLF14, LDB2, MEIS1-AS3, NKIRAS2, RPA2, SAMD10, TRIM59, and ZYG11A) in brain, bone, muscle, buccal swabs, and whole blood of 29 deceased individuals (0 to 87 years). Their analysis included a larger number of markers, which, although not ideal for a model applicable in forensic cases, could also offer an opportunity for further exploration of these methylation regions in subsequent research. The VISAGE Consortium also developed prediction models using samples from 24 deceased individuals, including blood, cartilage, and muscle. While the blood prediction model achieved a MAE of 3.1 years, further investigation is needed to improve the accuracy of cartilage and muscle samples, with respective MAEs of 13.1 and 17.1 [62].

The studies conducted so far have shown significant variability in techniques, the selection and combination of DNA methylation markers, and even in the time interval between sample collection and analysis. These variations could have influenced the accuracies obtained. However, it was also discovered that the difference in age prediction accuracy observed in samples from living and deceased individuals, as well as that observed in biological sex, may not be significant. These findings are particularly relevant in a forensic context.

#### 5.1.2. Y-Chromosome in Blood Samples

The study of DNA methylation in the Y-chromosome (ChrY) presents significant interest for forensic investigations [162,163]. Firstly, age prediction through DNA methylation could help in estimating the age of male individuals in mixed stains, such as those found in assault cases. Additionally, it could assist in distinguishing between male relatives of different ages within the same paternal lineage, a challenge not feasible with current ChrY analyses. However, this chromosome exhibits unique characteristics: it is exclusive to males, haploid in nature, and the smallest human chromosome. Consequently, a distinct approach is necessary in comparison to autosomal chromosomes.

In recent years, the earliest studies related to Y-chromosome and age prediction have been primarily focused on the different methylation patterns and their association with mortality [164], studying ChrY blood-based DNA methylation data from 624 men in a chromosome-wide epigenetic association analysis. They identified up to 416 CpG sites that exhibited differential methylation across ages. The results showed an increasing tendency in DNA methylation with age, a finding that was supported in further studies [163]. Additionally, later work from Lund et al. [165] found a significant overlap between mortality-associated and age-associated CpGs. Although these studies contributed to a deeper understanding of the ChrY and methylation patterns, they were not conducted with the aim of developing an age prediction model.

Vidaki et al. [163] developed the first male-specific Y-CpG-based epigenetic age predictor using publicly available blood-based DNA methylation data of 1057 European males (aged 15–87) obtained previously by Illumina HumanMethylation450 BeadChip array (San Diego, CA, USA). Machine learning was applied to create two age prediction models: one utilizing 75 age-dependent Y-CpGs (MAE = 7.54), and the other only employing the most predictive 19 Y-CpGs (MAE = 8.46). Although MAEs are higher compared to studies on autosomal chromosomes, this research sets a path for conducting further research for the application of Y-chromosome age prediction models in forensics.

In 2023, Jiang et al. [166] investigated 13 age-related Y-CpGs using publicly available DNA methylation data from 817 blood samples of males aged 15 to 87, obtained through the Illumina HumanMethylation450 BeadChip array (San Diego, CA, USA). They developed two SNaPshot systems for a male-specific age prediction model, achieving MADs between 4 and 6 years. Despite the moderate accuracy, this research holds promise for future studies. Additionally, the study incorporated the analysis of bloodstains as well as mixed samples.

Research on the Y-chromosome and its association with DNA methylation in age prediction remains limited. Further investigations of this entire chromosome, using non-array methodologies and focusing on microarrays of Y-CpG markers in non-blood tissues, are crucial for developing future prediction models relevant to forensic science applications [163].

### 5.2. Semen

Semen traces constitute a primary biological material for perpetrator identification in forensics, especially in cases of sexual assaults [167]. Furthermore, because of the unique age-related DNA methylation pattern observed in sperm cells compared to somatic cells [168,169], epigenetic clocks, such as Horvath’s [14], have not been able to accurately estimate age within this specific context.

In recent years, there has been a growing body of research dedicated to age estimation models in sperm cells. The studies so far have focused on identifying the most suitable age-correlated candidates using a combination of individual CpGs or broader regions known as DMSs (differentially methylated sites), which could introduce complexity to the marker selection process [114]. FOLH1B, located on chromosome 11, is a highly studied gene for semen age prediction, encoding folate hydrolase 1b, also known as prostate-specific membrane antigen-like protein. It has been researched for both age estimation in forensic contexts and its potential role in the development of prostate cancer [170]. 

Hwan Young Lee et al. [97] developed the first age prediction model using sperm samples for application in forensic science. Initially, an assessment was conducted on 485,000 CpG loci from 12 sperm donors (aged 20 to 59 years) using the Infinium HumanMethylation450 BeadChip array (Illumina, San Diego, CA, USA) and identifying 24 potential epigenetic age predictors. Subsequently, the final age prediction model using SNaPshot was developed based on the more strongly correlated methylation regions (TTC7B, FOLH1B/NOX4, and cg12837463), achieving a MAD of 5.4 for the validation test (n = 37). In a follow-up validation model, Lee et al. [171] analyzed both sample donors (n = 12) and forensic casework samples (n = 19), resulting in a MAD of 4.8 and 5.2, respectively. A primary highlight of the study was the inclusion of forensic casework samples, achieving reproducible results with less than 5 ng of bisulfite-converted DNA, a factor of particular significance for its potential implementation in forensic contexts. Individuals in their 20s and 50s showed distinct MADs of 2.9 and 7.2, respectively, indicating that, in line with findings from other tissue models, prediction sensitivity decreases with age progression. These two studies [97,171] highlighted the importance of increasing the number of samples to improve reliability. Later, Li et al. [106] developed a predictive age model utilizing two CpG sites in genes TTC7B and NOX4/FOLH1B, previously studied by a research group [111], using samples from Chinese males aged 21 to 54 years in a bisulfite pyrosequencing model. One notable aspect of the study was the utilization of different types of semen samples (liquid semen, fresh seminal stains, aged seminal stains, and mixed stains of semen and vaginal secretion), which resulted in MADs range between 3.8 and 4.3 years.

Recently, the VISAGE Consortium [114] designed a three-stage study to explore potential age predictors suitable for sperm cells. The study included identifying and validating novel age-correlated CpGs, as well as developing a prediction model based on the top candidates. First, they used 40 semen samples (24 to 58 years) in Infinium Methylation EPIC^®^ BeadChip arrays (Illumina, San Diego, CA, USA) to target approximately 850,000 CpGs and identified distinctive age-correlated DMSs suitable for age prediction. Building upon this, the ten most promising candidate CpGs, along with the three markers previously reported [111], were validated in an independent set of semen-derived DNA samples (n = 125) using targeted NGS assays. Finally, a prediction model was developed and further validated consisting of four novel (SH2B2, EXOC3, IFITM2, and GALR2) and one previously identified (FOLH1B) DNAm markers, achieving a MAE of 5.1 years in the testing set (n = 54). 

Other investigations concerning age prediction from semen have predominantly adopted a clinical perspective. For instance, Jenkins et al. [172] analyzed data from previous studies [169,173,174] to develop a statistical DNA methylation model employing the Infinium HumanMethylation450 BeadChip (Illumina, San Diego, CA, USA) (n = 329). The model was based on 51 genomic regions and reported a MAE of 2.4. The study used samples with diverse characteristics and found that age prediction could be achieved regardless of fertility status. Additionally, smokers showed a tendency towards elevated age profiles. In a recent study conducted by Pilsner et al. [175], two distinct age prediction models were constructed employing Infinium Methylation BeadChip and machine learning analysis: one utilizing individual CpGs (120 CpGs) and the other incorporating the entire DMRs (318 CpGs). The models exhibited less than 1% overlap in CpGs between them, suggesting a substantial pool of potential candidates for further investigation. These studies, although oriented to clinics, could aid in understanding the relationship between DNA methylation and aging, ultimately paving the way for potential future research projects in the forensic field.

Additional factors must also be considered regarding age prediction in sperm. Limitations include the decline in both sperm quality and quantity among the general population in recent decades, as well as variability in sperm count per individual [167]. Low sperm count can lead to an increase in non-spermatozoa cells in the sample, underscoring the need for further research into the differences between methylation profiles from purified sperm cells and whole semen samples [106,176]. Furthermore, there is the added complexity of researchers using diverse DNA markers and terminologies (DMR vs. CgG) for age prediction in semen samples. Finally, it is necessary to evaluate and validate the applicability of models in mixed samples, which may be encountered in sexual assault cases.

### 5.3. Saliva and Buccal Swabs

Saliva and buccal swab samples have been extensively investigated for DNA methylation-based age prediction methods. These samples are used in forensic contexts due to their simple and noninvasive collection from individuals. Moreover, they are frequently encountered at crime scenes, such as in cigarette butts and bottles [103]. However, a significant challenge comes from the cellular heterogeneity of these samples, where the proportions of leukocytes and epithelial cells vary depending on the sample type [177] and are further influenced by individual characteristics [178]. Bocklandt et al. developed the first saliva model using only three CpGs, achieving an average accuracy of 5.2, based on data obtained from Illumina HumanMethylation27 microarrays (San Diego, CA, USA) [179]. Following this, several studies have focused on differentiating tissue and cell types and developing accurate combined models independent of the sample type [37,63,178].

Hamano et al. [84] developed an age prediction model utilizing 263 saliva samples (1 to 73 years) employing MS-HRM and focusing solely on two markers in the genes ELOVL2 and EDARADD. They achieved a MAD of 6.25 years for the test set (n = 50). Additionally, they applied the same model to seven samples of cigarette butts, obtaining a MAD of 7.65 years. The difference in MAD was attributed to the limited number of samples. Years later, Oka et al. [86] used MS-HRM on 113 saliva samples (aged 20 to 50 years) to investigate the impact of ancestry on age prediction through methylation in the EDARADD and FHL2 genes, based on previous studies [83,84]. The differences they found in the methylation levels of Japanese and Indonesian participants led them to conclude about the importance of considering the population of origin in existing DNA methylation age prediction methods.

Eipel et al. [178] was the first to address the differential cell composition in saliva samples and its impact on age estimation. In the study, 55 buccal swabs were utilized to create an age prediction model based on three CpG sites (specifically, genes PDE4C, ASPA, and ITGA2B) using pyrosequencing. The model achieved a MAD of 7.03 for the validation test. Subsequently, two additional CpG markers specific to cell type (genes CD6 and SERPINB5) were incorporated to distinguish between leukocytes and epithelial cells, leading to MADs of 5.09 and 5.12 for two independent validation tests. This model, known as the “Buccal-Cell-Signature”, exhibited greater accuracy compared to the model without cell-type specific CpG markers. 

Hong et al. [37] developed an age prediction model based on 226 saliva samples using the SNaPshot method. Initially, they employed Illumina BeadChip array (San Diego, CA, USA) data from 54 individuals to identify the most age-associated CpG markers. Then, they utilized 226 saliva samples (aged 18 to 65 years) to construct an age prediction model based on six CpG markers (genes SST, CNGA3, KLF14, TSSK6, TBR1, and SLC12A5), along with one cell type-specific CpG marker (PTPN7 gene), in a SNaPshot assay. The testing set model (n = 113) achieved a MAE of 3.13. The incorporation of PTPN7 was based on its ability to distinguish between leukocytes and buccal epithelial cells. The use of a cell-specific marker was motivated by previous research [178]. A following study [96] investigated these same markers using both MPS technology and SNaPshot from saliva samples of the 95 individuals. As the predicted age obtained from these two methods varied greatly, they constructed platform-independent age predictive models, achieving a MAD of 3.19.

In a similar vein, 368 samples from 184 individuals (n = 184 saliva and n = 184 buccal cells) were analyzed using publicly available data from the Illumina HumanMethylation450 BeadChip array to select two tissue-specific markers (HUNK and RUNX1), along with seven age-correlated CpG sites (cg10501210, LHFPL4, ELOVL2, PDE4C, HOXC4, OTUD7A, and EDARADD). Subsequently, tissue-specific and combined age prediction models were developed using SNaPshot. The combined model, employing multivariate quantile regression, achieved a MAE of 3.66 on the testing set (n = 91 saliva and n = 93 buccal cells). In this case, no improvement was detected in age predictions when adding tissue-specific markers. Therefore, according to these results and those of previous studies [178], using markers of cellular composition as a co-variable was more effective than using tissue-specific markers [63].

Jung et al. [64] explored multiple tissues by analyzing five CpGs within the genes ELOVL2, FHL2, KLF14, C1orf132/MIR29B2C, and TRIM59. They developed independent and combined prediction models for saliva, buccal swabs, and blood samples using the SNaPshot assay. Specifically, in saliva samples (n = 150) and buccal swabs (n = 148), MADs of 3.55 and 4.29 were respectively obtained. In a separate study, Woźniak et al. [62] conducted MPS assays on various types of samples, including buccal cells. In this instance, they utilized five CpGs within the genes PDE4C, MIR29B2CHG, ELOVL2, KLF14, and EDARADD, achieving a MAE of 3.7 years in the testing set (n = 48).

Schwender et al. [65] developed age prediction models comparing pyrosequencing and SNaPshot. Initially, an analysis of 88 CpG sites in the genes PDE4C, ELOVL2, ITGA2B, ASPA, EDARADD, SST, KLF14, and SLC12A5 was conducted on buccal swab samples (n = 141) to identify markers that best correlated with age. Based on this, two prediction models were developed considering three markers in the genes SST, KLF14, and SLC12A5, one using pyrosequencing (MAD = 5.33) and the other using SNaPshot (MAD = 6.44). The aim was to compare the results of both methods, taking into consideration that SNaPshot could be more easily integrated into the routine workflow in a forensic laboratory.

One of the latest studies involves the first development of an age prediction model using ddPCR technology for human saliva samples. Initially, an analysis was conducted on methylation ratios at four CpG sites located within the genes SST, KLF14, TSSK6, and SLC12A5. Then, saliva samples from 76 individuals were employed to construct the prediction model, which yielded a MAD of 3.3 [118].

Koop et al. [105] investigated postmortem samples using buccal swabs from both living individuals (n = 142) and deceased individuals (n = 73), spanning ages from 0 to 90 years. Their goal was to develop an age prediction model based on a single gene, PDE4C, using a pyrosequencing assay. Methylation levels of PDE4C were assessed in the samples from deceased individuals at different stages of decomposition, and age estimation was not possible only in cases of advanced putrefaction. The main finding of the study was that DNA methylation remained stable across several stages of decomposition, and buccal swabs were suitable samples for assessing age-related methylation patterns in postmortem contexts.

In the context of forensic science, saliva and buccal swabs are among the most studied samples, alongside blood. The predominant focus in recent years has been on the type of sample to be analyzed and the technique used, resulting in varying approaches and impacting the accuracy of the different models.

**Figure 3:**
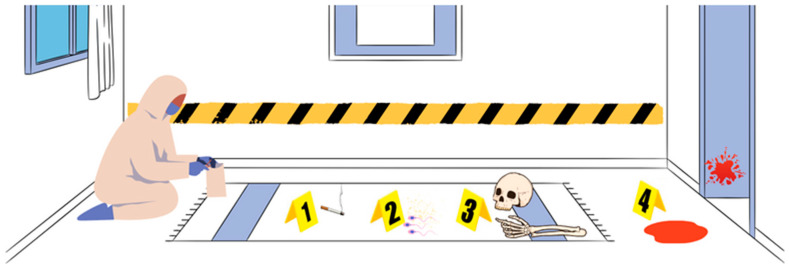
Possible biological samples found at the crime scene. The numbers of the evidence markers correspond to the ‘Sample Type’ column in Table 1.

The potential types of biological samples found at a crime scene and the accuracies of their age estimates based on DNA methylation are summarized in Table 1 and Figure 3.

### 5.4. Multi-Tissue Age Prediction Models

In forensic scenarios, distinguishing between various types of samples can be challenging. Therefore, the development of multi-tissue age prediction models would be highly beneficial. While tissue-specific models currently provide the most accurate age estimations [62], efforts are also directed toward creating universal markers suitable for multi-tissue samples. In this section, we will explore some of the multi-tissue models developed thus far.

Alsaleh et al. [180], identified 10 age-related DNA methylation markers and developed different age prediction models using samples from five tissue types: whole blood, saliva, semen, menstrual blood, and vaginal secretions. Their multi-tissue model, based on 41 samples, achieved an average prediction accuracy of 3.8 years in the training set. For the testing set (n = 24), three independent prediction models resulted in a MAD of 6.9 years for menstrual blood and vaginal fluid, 5.6 years for buccal swabs, and 7.8 years for blood. The overall multi-tissue accuracy rate, based on bootstrap analysis, was 7.8 years.

In another study, CpG markers previously studied [181] were examined in 29 samples from deceased individuals aged 0 to 87 years to explore the potential for developing a multi-tissue age predictor [38]. Utilizing massive parallel sequencing, the study revealed the capability of markers within 13 CpG regions of genes such as DDO, ELOVL2, KLF14, NKIRAS, RPA2, TRIM59, and ZYG11 previously studied [181] to predict age across various tissues including the brain, bones, muscles, buccal epithelial cells, and blood.

Alghanim et al. [182] examined 27 CpG sites within the SCGN, DLX5, and KLF14 genes across blood (n = 71) and saliva samples (n = 91) using pyrosequencing. Methylation levels at CpG sites within the SCGN and KLF14 loci were found to be correlated with chronological age in both tissues. Various predictive models were tested, ultimately resulting in age prediction with MADs ranging between 7.1 and 10.3 in independent testing datasets. 

In the study by Jung et al. [64] mentioned in previous sections, samples of saliva, blood, and buccal swabs showed strong correlations with age across three CpG sites within the genes ELOVL2, KLF14, and TRIM59. Three different tissue-specific models for age prediction and a combined model that included data from all three sample types were developed using SNaPshot, achieving a MAD of 3.8.

Also described previously, in the research from Correia Dias et al. [60], two multi-tissue age prediction models were the Sanger sequencing model and the SNaPshot assay. 

From the studies mentioned in this section, it can be observed that multi-tissue age prediction models currently demonstrate lower accuracies compared to those based on individual tissues. Therefore, it could be inferred that further analyses with different markers are necessary to reduce this disparity.

## 6. Other Factors Impacting DNA Methylation

In recent years, extensive research has delved into the impact of various exogenous and endogenous factors on DNA methylation patterns. These factors may contribute to differences between epigenetic age and chronological age, crucial in forensic casework, as it directly affects the accuracy of age estimation [183].

Spólnicka et al. [26,31] have shown differences in DNA methylation markers associated with age prediction in Alzheimer’s, Graves’ disease, and cancer, particularly chronic lymphocytic leukemia (CLL). Furthermore, infections like HIV [184], *Helicobacter pylori* [185], and cytomegalovirus [186] have been associated with increasing age prediction. Recent studies have suggested that both the COVID-19 virus and its medical management can impact DNA methylation levels at specific CpG loci, resulting in significant changes in epigenetic age clocks [134,187].

Additionally, research in forensic epigenomic profiling now is also focused on the advancement of predicting lifestyle habits like smoking, alcohol intake, diet, and sports [19,113,188,189,190,191]. For example, Spólnicka et al. [191] observed accelerated DNA hypermethylation in elite athletes, while healthy nutrition [133] was associated with decreased epigenetic age estimates. Conversely, insomnia [192] and working night shifts [193] were linked to increased age estimates (reviewed [183]).

The VISAGE Consortium recently conducted a study that explored the influence of alcohol intake on DNA methylation-based age prediction [113]. The study analyzed eight DNAm age predictors (ELOVL2, MIR29B2CHG, TRIM59, KLF14, FHL2, EDARADD, PDE4C, and ASPA) in individuals with alcohol dependency, using the VISAGE Enhanced Tool for age prediction previously described for somatic tissues [62]. Among these markers, MIR29B2CHG was the only one that showed an impact on age prediction, albeit a small one. Moreover, the study highlighted the need for further exploration of MIR29B2CHG, its function, and its connection with alcohol intake [113]. Vidaki et al. [188] further employed MPS technology to develop a new assay for exploring DNA methylation markers associated with smoking habits. Interestingly, out of the thirteen smoking-associated CpGs previously investigated by Maas et al. [190], eight CpGs were strongly correlated with age, with one [18] also showing an association with biological sex.

Adding further complexity, studies have demonstrated that DNA markers can vary depending on the ancestry. For example, differences in DNA methylation patterns were observed in specific genes (ELOVL2, FHL2, MIR29B2CHG, TRIM59, and KLF14) in blood samples from Polish [18], Korean [54,64], Italian [94], and Portuguese populations [95]. Inter-population differences in DNA methylation markers related to age prediction models have also been described in saliva samples [86] and semen samples [114]. This underscores the importance of ancestry analysis across different types of samples in age prediction through DNA methylation.

## 7. Concluding Remarks

Epigenetics, with a particular focus on DNA methylation, is currently a field of extensive study. It serves both aiding in age prediction within forensic contexts and contributing to research on aging, lifestyle, and diseases in the clinics. A central question in this field is whether the epigenome actively contributes to the aging process or if aging itself influences epigenetic patterns, leading to DNA methylation serving as an age marker. Advancing the understanding in this field is crucial for addressing this question and enhancing the development of epigenetic clocks and age prediction models.

Ensuring robust and reproducible results is imperative for incorporating different methods into criminal investigations. The diversity in techniques poses a potential barrier when comparing outcomes. Results obtained over the years suggest that both pyrosequencing and NGS would be the preferred technologies for further research. These methods have contributed to advancements in the analysis of DNA methylation and its correlation with age estimation, thereby improving accuracy and broadening knowledge in the field. While the equipment costs may be high, it is essential to acknowledge their high-quality price ratio. This consideration is important, given the significance of using methods that are accessible and affordable for forensic laboratories. Moreover, efforts could be directed towards enhancing bisulfite technique or exploring alternatives such as non-chemical conversions for the future. 

In the realm of epigenetic clocks, chronological clocks are most suitable for forensic purposes. Additionally, tissue-specific clocks (especially for semen, bones, and teeth, where information is scarcer) could play a crucial role in future enhancements. Ultimately, improving epigenetic clocks may offer insights into identifying the most efficient set of DNA markers for precise age prediction.

In anthropology, studies on bones and teeth are relatively limited compared to other tissues, and the results often exhibit highly variable accuracies. It is important to consider that the primary goal in this case is for DNA methylation to enhance the accuracy of classical anthropological techniques. Hence, the choice of the suitable technique and tissue is paramount. Thus far, pulp appears to be the tissue with the highest accuracy, and further studies employing the most advanced technologies could prove highly beneficial.

Blood samples have been the most extensively studied, both in epigenetic clock research and age prediction models in criminalistics. Various techniques and statistical models have been employed, as well as different approaches such as age estimation from bloodstains and samples from deceased individuals. Currently, there seems to be no difference in age prediction accuracy between living and deceased individuals. Furthermore, recent advancements have been made in the study of the Y-chromosome from blood samples, although studies are still limited, resulting in highly variable outcomes compared to somatic chromosome studies, and further research is needed in non-blood tissues. 

Saliva and buccal swab samples have also been widely studied. Their analysis using different techniques and sample types has contributed to improving accuracy over the years. Models have been examined in saliva, buccal swabs, and even in cigarette butts. Additionally, a deeper understanding of cellular heterogeneity has been achieved, which is a crucial consideration when working with any type of sample. Another example is semen, where the proportion of spermatic and non-spermatic cells may also impact age prediction. However, this tissue has been much less studied, and the consensus on which markers to use is not as clear-cut as in the case of saliva or blood samples. Additionally, new studies have been conducted on other types of tissues such as fingernails [194], hair [195], and menstrual blood [196].

In general, the authors of the aforementioned studies emphasize the importance of type and sample size in research, along with the selection of the best DNA methylation markers. The key objective is to use the fewest but most informative markers possible, making them more suitable for forensic use. Studies have also proposed multi-tissue models for age prediction, which, overall, have not yet demonstrated the accuracy observed in tissue-specific models, although they are considered ideal in forensic contexts.

Testing age prediction models across external and internal factors is crucial, as authors have identified influences on age prediction attributed to lifestyle and several conditions. Additionally, ancestry seems to impact DNA methylation patterns. Therefore, the authors stress the significance of conducting research across different regions globally or integrating samples with diverse origins into the same study.

There are studies to date made by scientific groups in collaboration to validate DNA methylation age prediction models, important for their future successful integration into standard forensic workflows [124,197]. Additionally, interdisciplinary research is expected. For example, aging has also been studied in relation to different ‘omics’ including transcriptomics [198], proteomics [199], and post-translational modifications such as glycans [200]. The interplay between them, alongside methylomics, holds promise for advancing our understanding of aging.

Future research endeavors should prioritize validating proposed methodologies and enhancing their accuracy. The translation of these techniques into the practice of forensic science and forensic anthropology necessitates fulfilling the requirements set by the Organization of Scientific Area Committees (OSAC) for their future application. Despite the challenges discussed, the remarkable advancements and evolution in epigenetics in recent years foster expectations that, with continued knowledge and discoveries, the ultimate application of age prediction in forensic sciences will be achieved in the near future.

## Figures and Tables

**Table 1:** Description of sample types, methods, and associated accuracies in recent publications. The numbers in the sample types correspond to the evidence markers in Figure 3.

Sample Type	Method	Accuracy in Years (Validation/Testing Set)	Authors
(1) Saliva	MS-HRM	MAD 6.25	[84]
	SNaPshot	MAE 3.13	[37]
		MAE 3.66	[63]
		MAD 4.29	[64]
	SNaPshot/NGS	MAD 3.19 (platform independent)	[96]
	ddPCR	MAD 3.3	[118]
(1) Buccal Swabs	SNaPshot	MAD 3.55	[64]
		MAD 6.44	[65]
	Pyrosequencing	MAD 5.33	[65]
		MAD 5.09–7.03	[178]
	NGS	MAE 3.7	[62]
(1) Cigarette Butts	MS-HRM	MAD 7.65	[84]
(2) Semen	SNaPshot	MAD 5.4	[97]
		MAD 4.8–5.2	[171]
	Pyrosequencing	MAD 3.8–4.3	[106]
	NGS	MAE 5.1	[114]
		MAE 2.4	[172]
(3) Teeth	Pyrosequencing	MAD 4.86 (dentin)	[57]
		MAE 1.5–2.13 (pulp)	[59]
	EpiTYPER	MAD 1.2–7.1 (dentin, pulp, cementum)	[58]
	Sanger sequencing	MAD 11.35	[75]
	RT-MSP	MAD 8.94	[77]
		MAE 6.69–8.28	[78]
	SNaPshot	MAD 7.1	[75]
	Pyrosequencing	MAE 4.8–6.9	[108]
(3) Bones	Sanger sequencing	MAD 2.56	[75]
	SNaPshot	MAD 7.2	[75]
	NGS	MAE 3.4	[62]
(4) Blood	EpiTYPER	MAD 2.49	[88]
	SNaPshot	MAD 3.48	[64]
		MAD 5.56	[93]
		MAD 3.01	[94]
	Pyrosequencing	MAD 4.5	[15]
		MAD 5.75	[67]
		SE 3.9	[18]
		MAD 3.29	[54]
	NGS	MAD 3.76 (statistical analysis from array data)	[110]
		MAE 3.2	[158]
		MAE 3.2	[62]
		MAD 2.8–2.93 (Bloodstains)	[56]
(4) Postmortem Blood	Sanger sequencing	MAD 8.84 (Deceased individuals)	[161]
	MS-HRM	MAD 7.71 (Living and deceased individuals)	[83]
	SNaPshot	MAD 4.25 (Living individuals)	[95]
		MAD 5.36 (Deceased individuals)	
		MAD 4.97 (Living and deceased individuals)	
	Pyrosequencing	MAD 3.75 (Living and deceased individuals)	[57]
		MAD 7.42	[104]
	NGS	MAE 3.1 (Deceased individuals)	[62]
(4) Blood (ChrY)	SNaPshot	MAD 5.73	[166]
	NGS	MAE 7.54–8.46	[163]

## Data Availability

No new data are reported in this review article.
